# Bone erosion in the 2nd metacarpophalangeal head: association with its bone mineral density by HR-pQCT in rheumatoid arthritis patients

**DOI:** 10.1186/s12891-021-03992-5

**Published:** 2021-01-25

**Authors:** Camille P. Figueiredo, Mariana O. Perez, Lucas Peixoto Sales, Ana Cristina Medeiros, Valeria F. Caparbo, Rosa M. R. Pereira

**Affiliations:** 1 Bone Metabolism Laboratory, Rheumatology Division, Faculdade de Medicina FMUSP Universidade de Sao Paulo, Av. Dr. Arnaldo 455, 3° andar, sala 3105, Sao Paulo, 01246-903 Brazil; 2 Rheumatology Division, Hospital das Clinicas HCFMUSP, Faculdade de Medicina FMUSP Universidade de Sao Paulo, Sao Paulo, SP Brazil

**Keywords:** HR-pQCT, Bone erosions, Periarticular bone loss

## Abstract

**Background:**

Rheumatoid arthritis (RA) is a chronic autoimmune disease depicted by synovial inflammation leading to local and systemic bone loss. The aim of this study was to evaluate by a HR-pQCT (High Resolution Peripheral Quantitative Computed Tomography) study which parameters are associated with volume of bone erosions including bone mineral density (BMD) around erosions (VOI 1 to 4 = volume of interest), BMD of metacarpophalangeal (MCP) head, BMD of radius, presence of osteophytes and joint space width (JSW).

**Methods:**

Fifty female RA patients (18–50 years) were enrolled in this study. Demographic and disease-specific data, laboratory inflammatory parameters and handgrip test were performed. All patients underwent HR-pQCT of 2nd and 3rd MCP joints and distal radius, according to established protocols. The volume of bone erosions was evaluated by MIAF (Medical Image Analysis Framework) software. Osteophytes were analyzed by manual method.

**Results:**

The mean of age and disease duration were 40.0 ± 6.0 yrs. and 10.8 ± 4.8 yrs., respectively. According to DAS-28 (Disease Activity Score), 54% (27) of the sample were in remission. However, when SDAI (Simplified Disease Activity Index) was used, only 18% (9) were under remission. The mean of HAQ (Health Assessment Questionnaire), ESR (Erythrocyte sedimentation rate) and CRP (C reactive protein) were 0.9 ± 0.7, 13.9 ± 12.2 mm and 5.6 ± 7.5 mg/mL, respectively. Forty-six bone erosions (0.9 ± 1.2 erosion/patient) and 14 osteophytes (0.3 ± 0.7 osteophyte/patient) were found in 2nd MCP head. The median (IQR-Interquartile range) of volume of erosion and volume of osteophytes were 14.9 (5.7;35.9)mm^3^ and 3.1 (2.1, 4.3)mm^3^, respectively. The mean of JSW was 80.5 ± 34.2 mm^3^. The volume of bone erosions was negatively correlated with BMD of 2nd MCP head, VOI-4 and JSW; and it was positively correlated with osteophytes number. Regarding absence or presence of erosion in 2nd MCP head, a significant difference was found between BMD of MCP head, osteophyte number and JSW. Multiple linear regression analysis showed that only BMD of 2nd MCP head was independently associated with volume of bone erosions.

**Conclusion:**

BMD of MCP head was independently associated with volume of bone erosion, suggesting that this parameter should be used to analyze and monitoring bone destruction, as well as to evaluate treatment response in RA patients.

## Background

Rheumatoid arthritis (RA) is a chronic inflammatory disease characterized by persistent synovial inflammation and bone loss that might lead to progressive joint damage, especially in small joints of hands and feet, deformities and functional disability [1]. Bone damage in RA is depicted by bone erosions, and these destructions depends on many features as autoantibodies, tight treatment control and disease duration [2].

It is also well established that patients with RA develop both local and systemic bone loss, which is mainly mediated by inflammation [3], leading to an increased fracture risk independently of glucocorticoid use [4]. During early stages of RA, the disease gradually develops joint swelling, stiffness and pain, besides that patients often have a history of long timing symptoms when first presenting to a rheumatologist. Periarticular demineralization of metacarpal bones may already be present at this stage, representing an early radiologic manifestation visible on plain radiographs [5, 6], with a closed association to inflammatory activity in RA [7]. However, data on quantifying periarticular bone loss in RA patients is not precise when standard techniques are used, as plain radiography [8, 9] or dual- energy X-ray absorptiometry (DXA), restricted to areal bone mineral density (aBMD), validated for femur and spine [10, 11].

 High-resolution peripheral quantitative computed tomography (HR-pQCT), is an in vivo clinical imaging technique, enable to assessment bone microarchitecture quantifying trabecular and cortical bone in radius and tibia [12]. In addition, this technique arises as an important tool to bone structure analysis in inflammatory bone diseases, mainly in RA patients, especially because of its capability in detecting precise dimensions of even small erosions or bone proliferation in metacarpophalangeal (MCP) head [13, 14]. Until now, there are only a few published articles using this technology to evaluate focal bone loss in RA patients, and none of them compared periarticular BMD with volume of bone erosions [15–17]. Besides, it has been proposed that erosions healing starts by bone apposition at the bottom of the lesions, so that the BMD around erosion should be more related to volume of erosions than total metacarpal head BMD, and osteosclerosis could represent healing of erosions [18].

In this way, the aim of this study was to evaluate which parameters are associated with volume of bone erosions including BMD around erosions, BMD of 2nd metacarpophalangeal head, BMD of radius, presence of osteophytes and joint space width.

## Methods

### Subjects

Fifty female RA patients were selected to the present study from the Rheumatoid Arthritis Outpatient Clinics from Hospital das Clinicas, Faculdade de Medicina, Universidade de São Paulo, São Paulo-Brazil. All patients between 18 and 50 years who fulfilled the inclusion criteria and accepted to participate were enrolled in this study. The local ethics committee approved the study and all participants read and signed the informed consent for the study.

Demographic and disease-specific data (treatment, disease duration, disease activity) were collected, as well as laboratory specific measures were performed: erythrocyte sedimentation rate (ESR) and C reactive protein (CRP). Swollen joint count-28, tender joint count-28, visual analogue scale for patient and physician global disease activity and visual analogue pain scale were assessed by an experient rheumatologist (MOP), in order to determine DAS-28 (disease activity score) and SDAI (simplified disease activity index).

### Exclusion criteria

Exclusion criteria were other associated autoimmune diseases, postmenopausal women, current pregnancy, kidney failure, uncontrolled diabetes, malabsorption diseases, other diseases of the bone metabolism (hyperparathyroidism, Paget’s disease and bone dysplasia), neoplasms solid and hematological, and use of bisphosphonates at the current time or in the last year.

### HR-pQCT acquisition

**Fig. 1:**
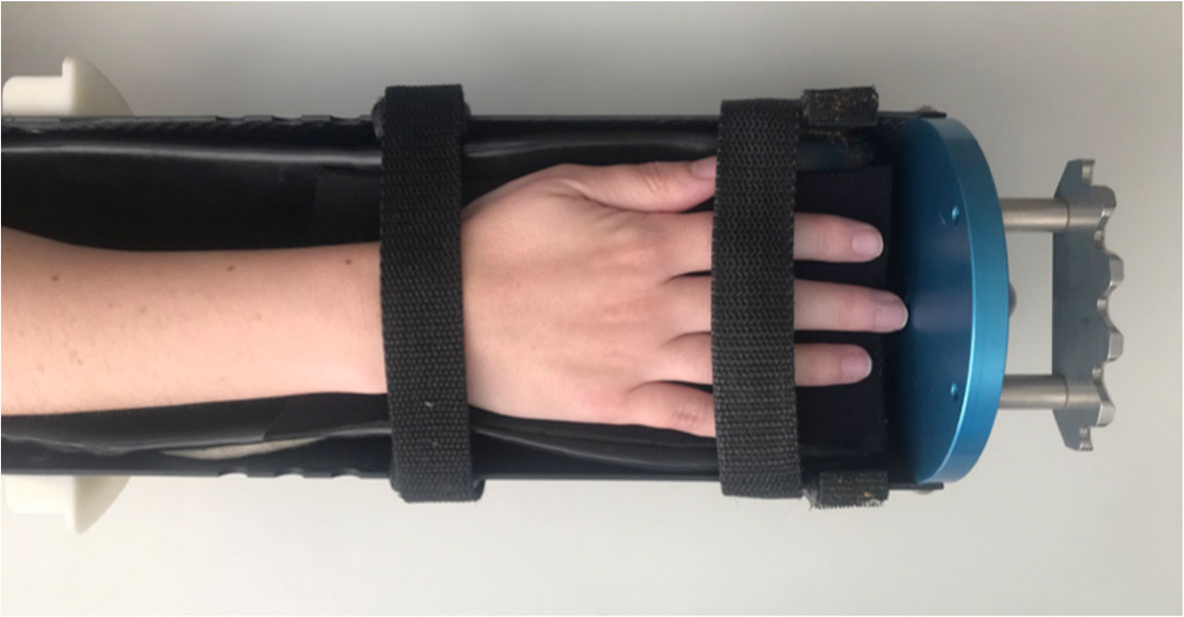
Photograph of the hand cast used in Bone Metabolism Laboratory, Rheumatology Department, Faculdade de Medicina, University of São Paulo. A palm down orientation is used to evaluate 2nd and 3rd MCP (metacarpophalangeal) and PIP (Proximal Interphalangeal) joints

All patients underwent scans of distal radius from non-dominant side, and scans of 2nd and 3rd MCP joints of the dominant hand, according to SPECTRA group established protocol [19]. Image acquisition was performed using an XtremeCT first generation scanner from Scanco Medical (Scanco Medical AG, Brüttisellen, Switzerland). Radius scans were performed with a stack of 110 parallel CT slices with an isotropic voxel size of 82 × 82 × 82 mm. For the assessments, the patient’s non-dominant forearm was positioned in the thumb-up position according to the manufacturer’s standard in vivo protocol [20]. For MCPs analysis, the dominant hand was positioned in an outstretched prone position and padded (Fig. 1). The same custom-made holder was used for all patients’ assessments. Scanning was performed within 80 slices distal to and 242 slices proximal to the surface of the third metacarpal head. Motion grade artefacts were characterized to both radius and MCPs analysis, from grades 1 to 5 according to literature [21], and only grades 1 to 3 were used, in case of grades 4 and 5, the scan was repeated.

### Distal radius analysis

The images were segmented and processed using a standard protocol (Scanco Medical AG, Brüttisellen, Switzerland) [20]. First, a semi-automated segmentation is applied to the original grayscale image to contour the periosteal surface. After that, the cortical and trabecular regions were segmented automatically by the analysis protocol and then densitometric and structural parameters from HR-pQCT images became available as following: Tt.vBMD (total volumetric bone mineral density), Tb.vBMD (trabecular volumetric bone mineral density) and Ct.vBMD (cortical volumetric bone mineral density), as well as Tb.N (trabecular number), Tb.Th (trabecular thickness), Tb.Sp (trabecular separation) and Ct.Th (cortical thickness).

### Peripheral bone changes assessment (erosions and osteophytes)

In order to precisely achieve the relationship between volume of bone erosion with local BMD, in this study we considered only the analysis of 2nd metacarpal heads. However, the scans were done evaluating both 2nd and 3rd MCPs of dominant hand in all patients. For specific site of bone lesions, the metacarpal head was divided into 4 quadrants (I = palmar, II = ulnar, III = dorsal and IV = radial). Erosions were defined as breaks in the cortical bone shell, which were visible in at least 2 planes, and osteophytes were defined as bony protrusions emerging from the juxta-articular cortical shell that were visible in at least 2 planes [22]. The number of bone lesions in each quadrant was counted. MCP heads with more than one erosion or osteophyte, only the size of the largest lesion was measured.

**Fig. 2:**
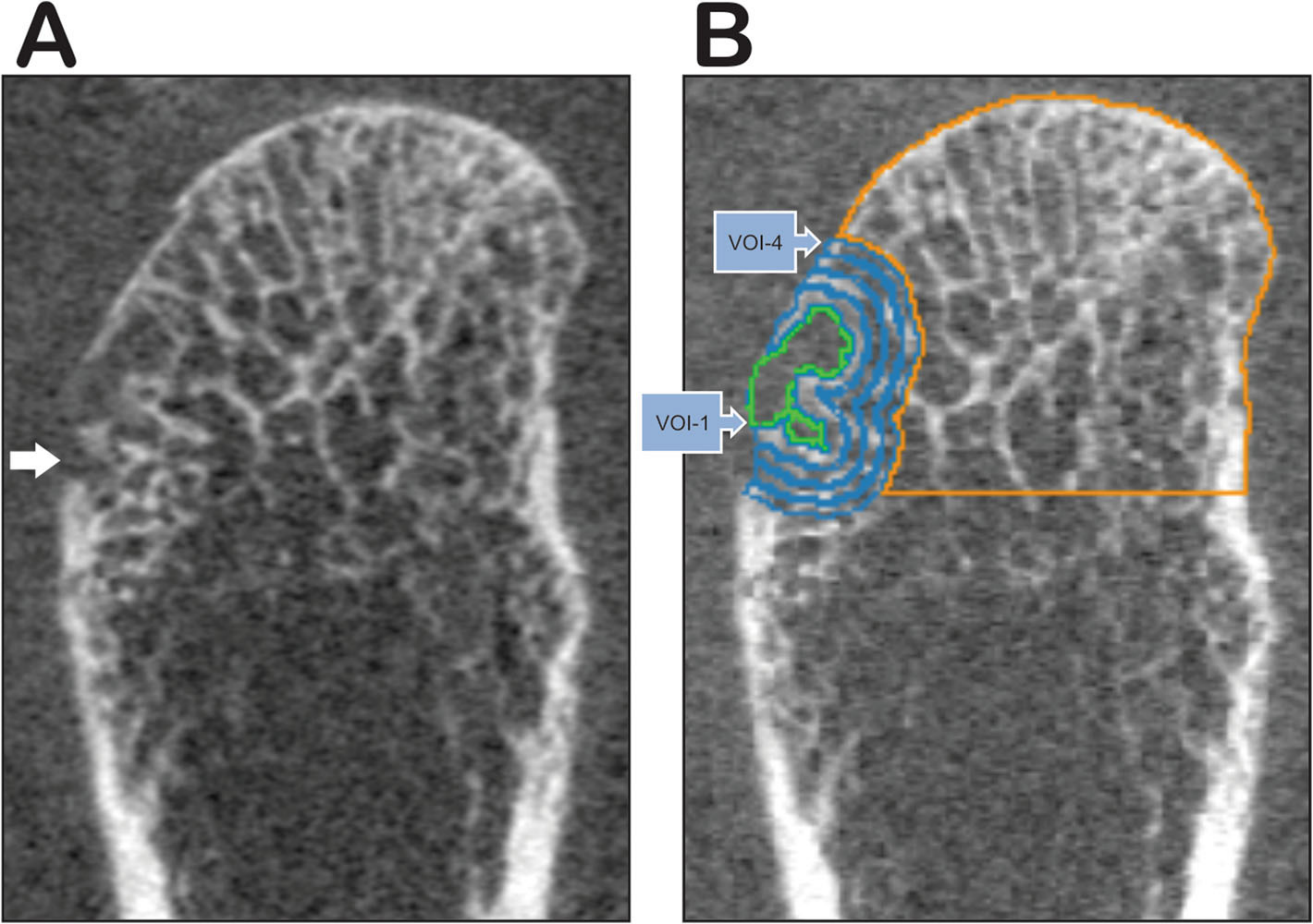
HR-pQCT imaging of 2nd metacarpophalangeal head (MCP) from RA patient in a coronal plane. **a** Bone surface of the metacarpal head with an erosion (white arrow). **b** erosion Demonstration of bone segmentation by Medical Image Analysis Framework software (MIAF): bone mineral density (BMD) of MCP analyzed in orange line; erosion segmentation placed on radial quadrant (quadrant IV) in green line; and around the analyzed erosion 4 layers of segmented BMD (Volume of Interest: VOI) in blue lines

The erosion volume was evaluated by semi-automated software named MIAF (Medical Image Analysis Framework) that applies a full 3D segmentation of erosions, previously published [14, 23]. Briefly, MCP segmentation is made and the periosteal surface ‘closes’ the cortical breaks, then the operator places a seed point in each lesion. Then, the erosion is segmented using the level-set method, and inflating a spherical structure centered at the seed point, which stops at trabecular bone, at the same time the periosteal segmentation is used as the edge of the analyzed erosion, after that some manual correction might be assembled by the user if necessary. Besides the erosion volume, MIAF automatically gives the total BMD of metacarpal head, BMD around analyzed erosions, separated by 4 layers [VOI (volume of interest) 1 to 4], both results are given in mg/cm^3^ (Fig. 2), and the joint space width, expressed in mm^3^.

Osteophytes were analyzed according to the manual method, previously described [13]. Briefly, after the open-source Digital Imaging and Communication in Medicine viewer (OsiriX software, version 7.5) assessed the images, the osteophytes were considered geometrically as a cone with an axial ellipsoid base. Then, the ellipsoid area multiplied by a perpendicular height of the osteophyte and divided by 3 accounts for the volume in mm^3^.

### Statistical analysis

The data analysis was performed using IBM SPSS software, version 22.0. Gaussian distribution was tested by the Kolmogorov-Smirnov test. All data are expressed as mean and standard deviation (SD), number (%) or median and interquartile range (IQR), as appropriate. Spearman’s correlation test was applied in order to assess the interdependence of structural and densitometric parameters. Presence or absence of erosions in 2nd MCP head subgroups was compared using Mann-Whitney test. A multiple linear regression was calculated using the stepwise forward method, considering *p* < 0.05 as level of inclusion and the volumetric trabecular BMD of radius (representative of systemic bone loss), with volume of erosion used as a dependent variable. *P* values less than 0.05 were considered significant.

## Results

In this cross-sectional study, 50 female patients with established RA were enrolled. All demographic, clinical and laboratory parameters are described in Table 1. When DAS-28 is considered, 54% (*n* = 27) of all patients were under remission, on the other hand, when SDAI analysis is used, remission is found in only 18% (9) patients. The mean of handgrip evaluation was 18.0 ± 7.3 N, which is accountable for lower strength compare to healthy women at the same age (around 28 N) [24]. Regarding the current treatment during the study, 62% (31) of all analyzed women were using glucocorticoids, 94% (47) received conventional Disease Modifying Anti-Rheumatic Drugs (DMARDs) and 43% (21) were in treatment with biological DMARDs.

**Table 1:** Demographic and clinical characteristics of the RA patients enrolled in the study

Variables	***N*** = 50
**Clinical parameters**	
Age. years	40.0 ± 6.0
Disease duration. Years	10.8 ± 4.8
Disease activity score-28 (DAS-28)	2.7 (2.1; 3.1)
Remission. N(%)	27 (54%)
Mild disease activity. N(%)	5 (10%)
Moderate disease activity. N(%)	17 (34%)
High disease activity. N(%)	1 (2%)
SDAI	10.9 ± 8.2
Remission. N(%)	9 (18%)
Mild disease activity. N(%)	18 (36%)
Moderate disease activity. N(%)	20 (40%)
High disease activity. N(%)	3 (6%)
Hand Grip. N	18.0 ± 7.3
Current Treatment	
Glucocorticoids. N(%)	31 (62%)
conventional DMARDs. N(%)	47 (94%)
biological DMARDs. N(%)	21 (43%)
HAQ	0.9 ± 0.7
**Laboratory Parameters**	
ESR. mm/h	13.9 ± 12.2
CRP. mg/L	5.6 ± 7.5

The results are expressed as mean ± standard deviation (SD). mean (IQR = interquartile range) or N(%). *SDAI* Simplified disease activity index, *DMARD* Disease Modifying Anti-Rheumatic Drugs, *HAQ* Health assessment questionnaire, *ESR* Erythrocyte sedimentation rate. *CRP* C reactive protein

In order to correlate the BMD around erosive lesions with volume of these erosions with more precision, considering that the great majority of all bone lesions in the hand are found at the 2nd MCP joints, it was analyzed here only data of the 2nd MCP head. Forty-six erosions (0.9 ± 1.2 erosion per patient), with an erosion volume median (IQR) of 14.9 (5.7; 35.9) mm^3^ were found in the 2nd MCP head. It was also found 15 osteophytes (0.3 ± 0.7 osteophyte per patient), with a volume median (IQR) of 3.1 (2.1; 4.3) mm^3^. The joint space width was 80.5 ± 34.9 mm^3^. Descriptive data of all BMD values are described in Table 2.

**Table 2:** Bone mineral density values of all HR-pQCT analyzed sites

HR-pQCT Site	BMD
**2nd MCP head (number of patients = 50). mg/cm**^**3**^	302.6 ± 56.6
**Layers around erosion (number of patients = 26). mg/cm**^**3**^	
VOI-1	393.8 ± 81.2
VOI-2	347.7 ± 91.7
VOI-3	321.8 ± 78.3
VOI-4	304.9 ± 79.0
**Radius (number of patients = 49). g/cm**^**3**^	
Ct.vBMD	868.8 ± 64.4
Tb.vBMD	152.7 ± 40.3

The results are expressed as mean ± standard deviation (SD). *BMD* Bone Mineral Density, *Ct.vBMD* Cortical volumetric bone mineral density, *Tb.vBMD* Trabecular volumetric bone mineral density, *VOI* Volume of Interest

**Fig. 3:**
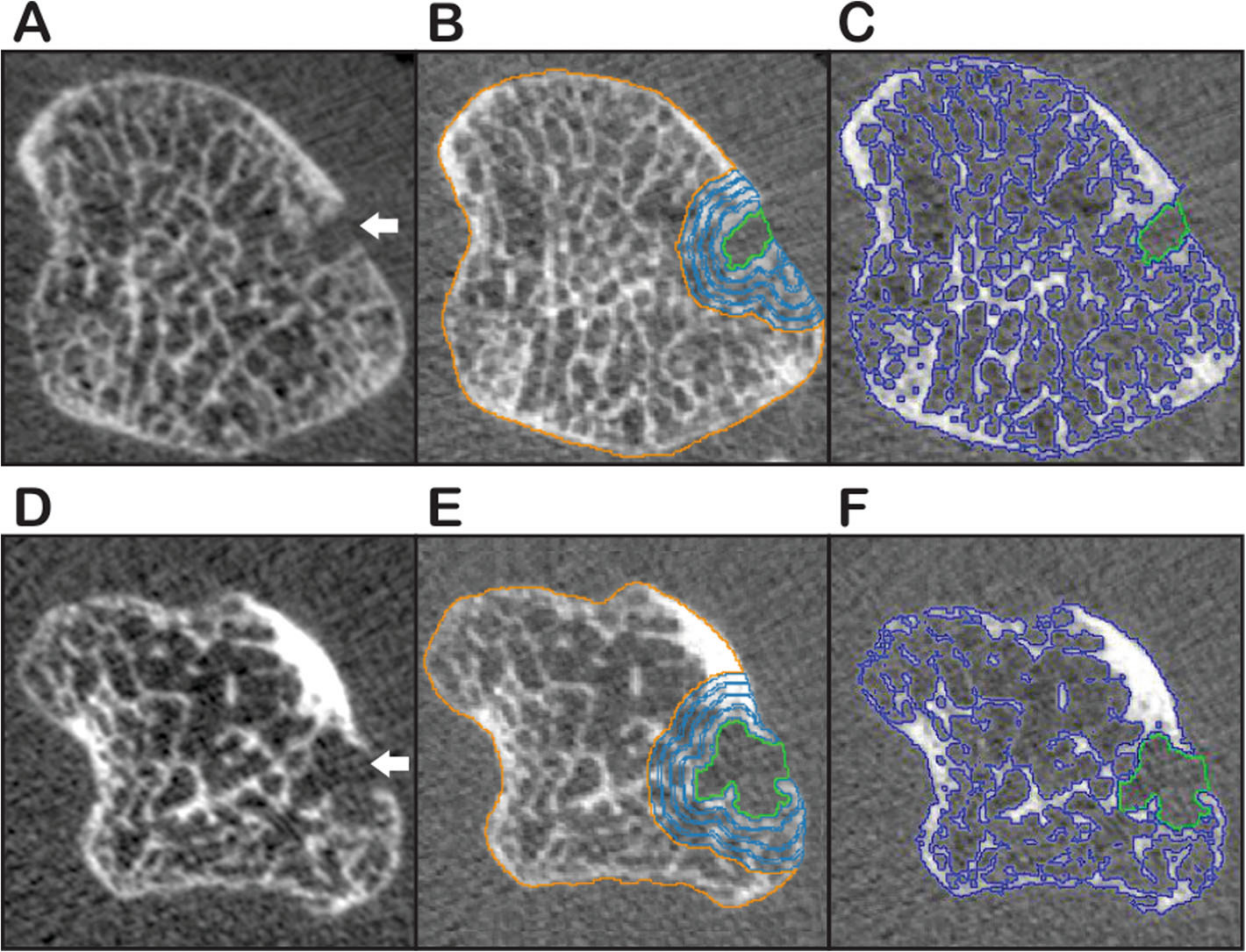
Axial plane of 2nd metacarpophalangeal head (MCP) from RA patients using HR-pQCT scan comparing two individuals with erosion on radial quadrant (quadrant IV). First patient (**a, b** and **c**): **a** metacarpal head with no segmentation, showing the bone erosion (white arrow); **b** Segmented erosion with volume = 6.75 mm^3^ with 4 layers of segmented bone mineral density (BMD) (Volume of Interest: VOI) in blue lines, and orange line represents BMD of MCP analyzed; **c** demonstration of evaluated BMD of MCP in purple contouring, with total amount of 314.94 mg/cm^3^. Second patient (**d**, **e** and **f**): **d** Metacarpal head with the bone erosion with no segmentation (white arrow); **e** Segmentation of a bigger erosion, volume = 19.33 mm^3^, besides 4 layers of segmented BMD in blue lines and **f** MCP segmented in purple contouring, that represents a BMD = 284.63 mg/cm^3^

Not surprisingly, the volume of bone erosions were significantly correlated with number of erosions (r = 0.82, *p* < 0.001). Moreover, volume of bone erosions was negatively correlated with BMD in 2nd MCP head (r = − 0.53, *p* < 0.001), VOI-4 (r = − 0.48, *p* = 0.017) and joint space width (r = − 0.40, *p* = 0.005) and positively correlated with number of osteophytes (r = 0.32, *p* = 0.026). Furthermore, the sample was categorized by absence or presence of erosion in 2nd MCP head (Table 3) and a significant difference was found regarding to BMD of 2nd MCP head (321.5 ± 37.1 vs. 281.3 ± 67.6 mg/cm^3^, *p* = 0.043) (Fig. 3), number of osteophyte (0.1 ± 0.4 vs. 0.5 ± 0.8, *p* = 0.028) and joint space width (93.5 ± 36.9 vs. 68.2 ± 28.3, *p* = 0.025).

**Table 3:** The sample was categorized by the presence or not of bone erosions in 2nd MCP head

Variables	Absence of Erosion (***N*** = 24)	Presence of Erosion (***N*** = 26)	P
**Age,** years	39.7 ± 6.3	40.6 ± 5.5	0.594
**Disease duration,** years	10.7 ± 5.3	11.2 ± 4.4	0.831
**DAS-28**	2.5 ± 1.3	2.6 ± 0.8	0.815
**SDAI**	12.8 ± 8.8	10.2 ± 8.0	0.271
**HAQ**	1.0 ± 0.7	0.7 ± 0.6	0.271
**Hand Grip, N**	18.6 ± 7.8	17.6 ± 6.5	0.670
**Number of Osteophyte**	0.1 ± 0.4	0.5 ± 0.8	0.028^a^
**BMD of MCP head,** mg/cm^3^	321.5 ± 37.1	281.3 ± 67.6	0.043^a^
**Joint space width,** mm^3^	93.5 ± 36.9	68.2 ± 28.3	0.025^a^
**Radio**			
Ct.vBMD, mg HA/cm^3^	875.1 ± 58.7	865.5 ± 70.1	0.602
Tb.vBMD, mg HA/cm^3^	147.4 ± 39.1	155.6 ± 43.6	0.109
**Tibia**			
Ct.vBMD, mg HA/cm^3^	925.7 ± 33.3	905.2 ± 49.5	0.215
Tb.vBMD, mg HA/cm^3^	139.2 ± 32.2	149.2 ± 34.6	0.382

The results are expressed as mean ± standard deviation (SD). *DAS-28* Disease activity score 28, *SDAI* Simplified disease activity index, *HAQ* Health assessment questionnaire, *BMD* Bone mineral density. *MCP* Metacarpophalangeal, *Ct.vBMD* Cortical volumetric bone mineral density, *Tb.vBMD* Trabecular volumetric bone mineral density. ^a^Statistical significance

A multiple linear regression was performed using volume of bone erosion as dependent variable, and as independent variables it was used number of erosions, number of osteophytes, joint space width, BMD of 2nd MCP head, VOI-4 and volumetric trabecular BMD of radius. The multivariate analysis found that BMD of 2nd MCP head was the only variable associated with volume of bone erosion (B = − 0.81, *p* = 0.003, adjusted R^2^ = 0.32).

## Discussion

Our study demonstrated that BMD of 2nd MCP head was the only variable independently associated with erosion size, showing that bone mineral density of total MCP head is rather more important than BMD around erosions (presence or absence of osteosclerosis at the bottom of some erosive lesions), BMD at distal radius, presence of osteophytes and joint space width.

Many studies in RA patients have demonstrated an association between bone erosions and the development of local and systemic bone loss, as osteopenia and osteoporosis [25, 26]. It is also well known that this periarticular osteopenia is the earliest radiographic change in RA patients, prior to the development of erosions [5]. However, the exactly site of joint related to bone erosion has not yet been evaluated and quantified. Black et al. [11] identified patients who were at risk of developing erosive disease at 12 months studying total hand BMD by DXA and erosions through hand and feet radiographs, scored using van der Heijde modification of the Sharp method. However, DXA method evaluates areal BMD, and HR-pQCT is able to analyze volumetric BMD. Moreover, it has been demonstrated that HR-pQCT scans allow detailed identification of articular bone damage [22, 27]. Besides that, through MIAF, the software we used here for bone erosions analysis, it was possible quantifying precisely both bone erosions in cubic millimeters (mm^3^) and periarticular bone loss [20, 28], as well as focal BMD around erosions [14]. Digital x-ray radiogrammetry is another method frequently used in many studies to assess hand or MCP BMD in arthritis patients, showing a good correlation between local bone loss and disease progression, but this still represents a semiquantitative method [7, 29, 30].

On the other hand, perilesional bone has been associated with erosion progression, and findings of osteosclerosis in this region might represent healing of erosions, even though these outcomes were evaluated according to semiquantitative scores, suggesting that BMD around erosions could be more associate with the volume of destructive lesions than BMD in other sites [31, 32].

Bone damage arises early in the course of RA [33]. Currently, the treat-to-target main purpose of RA management is based on prompt detection and control of erosion size, which represents directly the local amount of bone destruction. After the introduction of biologic therapies, precise procedures have been required to recognize parameters that could be associated with bone damage. In fact, this is one of the main purposes at RA studies, since it could allow an identification and selection of patients for more intensive therapy, including earlier addition of biologic agents, in order to limit severity and disease progression. Furthermore, it has been shown that routine disease activity biomarkers are not good predictors for radiologic progression damage in RA patients [34, 35]. Antibodies to cyclic citrullinated peptide (anti-CCP), CRP, other inflammatory markers levels, and initial radiographic damage are currently used to distinguish patients at greatest risk for increasing progressive bone destruction, in order to achieve aggressive treatment strategies in designated group of RA patients [36, 37]. Additionally, despite the advent of biologic agents, RA remains leading to joint destruction in most of the patients. In this way, identifying parameters that might be used for prediction of patients at risk of erosion progression is the target of most studies. Thus, the results of this study can be used to speculate that, in longitudinal investigations, it might be possible to identify earlier structural bone changes as periarticular bone loss, distinguishing patients who have a rapid radiographic progressive erosive RA from those with more insidious disease.

However, our study is not without limitations, as the size of the sample, as well as the analysis of only women. Nonetheless, RA is a disease typical found in female. Another limitation is the fact that both machine (HR-pQCT) and software (MIAF) are not widespread used, being found mainly in research centers. Further longitudinal studies may be needed, with both male and female genders to extend the findings.

Summarizing, our data showed that volume of erosions are closely and negatively associated with periarticular bone mineral density. Even when different BMD sites were analyzed as distal radius or BMD around erosions, periarticular bone loss remained the most important variable associated to the amount of bone destruction. Because detection of erosion size is an important goal in tracking RA progression and evaluating therapeutic reaction, our paper suggests that BMD of MCP head could be a helpful parameter to analyze and monitoring bone destruction, as well as to evaluate treatment response in RA patients.

## Data Availability

The datasets analyzed during the current study are available from the corresponding author on reasonable request.

## Abbreviations

RA: Rheumatoid Arthritis
HR-pQCT: High Resolution Peripheral Quantitative Computed Tomography
BMD: Bone Mineral Density
VOI: Volume of Interest
MCP: Metacarpo-phalangeal
JSW: Joint Space Width
MIAF: Medical Image Analysis Framework
DAS: Disease Activity Score
SDAI: Simplified Disease Activity Index
HAQ: Health Assessment Questionnaire
ESR: Erythrocyte Sedimentation Rate
CRP: C Reactive Protein
IQR: Interquartile Range
DXA: Dual- energy X-ray absorptiometry
aBMD: Areal Bone Mineral Density
Tt.vBMD: Total volumetric bone mineral density
Tb.vBMD: Trabecular volumetric bone mineral density
Ct.vBMD: Cortical volumetric bone mineral density
Tb.N: Trabecular number
Tb.Th: Trabecular thickness
Tb.Sp: Trabecular separation
Ct.Th: Cortical thickness
SD: Standard deviation
DMARDs: Disease Modifying Anti-Rheumatic Drugs
anti-CCP: Antibodies to cyclic citrullinated peptide
